# Trauma Patients and Acute Compartment Syndrome: Is There an Ariadne’s Thread That Can Safely Guide the Anesthesiologist/Emergency Physician Out of the Labyrinth?

**DOI:** 10.3390/medicina60081279

**Published:** 2024-08-08

**Authors:** Eleftheria Soulioti, Marianthi Pertsikapa, Barbara Fyntanidou, Pantelis Limnaios, Tatiana Sidiropoulou

**Affiliations:** 1 Second Department of Anesthesiology, Attikon University Hospital, National and Kapodistrian University of Athens, 12462 Athens, Greece; eleftheriasoulioti@ymail.com (E.S.); panlimn@hotmail.com (P.L.); 2 Department of Emergency Medicine, AHEPA University Hospital, 54636 Thessaloniki, Greece; marianpertsis@gmail.com (M.P.); fyntanidou@auth.gr (B.F.)

**Keywords:** acute compartment syndrome, pain management, peripheral nerve blocks, trauma patient

## Abstract

Trauma patients in the emergency department experience severe pain that is not always easy to manage. The risk of acute compartment syndrome further complicates the analgesic approach. The purpose of this review is to discuss relevant bibliography and highlight current guidelines and recommendations for the safe practice of peripheral nerve blocks in this special group of patients. According to the recent bibliography, peripheral nerve blocks are not contraindicated in patients at risk of acute compartment syndrome, as long as there is surveillance and certain recommendations are followed.

## 1. Introduction

Trauma patients in need of emergency care at the emergency department (ED) are suffering from orthopedic injuries in the majority of cases. Saving human lives is definitely the main priority, and under this prism, pain is not considered a priority. As a consequence, pain is often underestimated, misdiagnosed and undertreated. Due to the imminent risk of acute compartment syndrome (ACS) in this clinical setting, regional anesthesia (RA) and peripheral nerve blocks (PNBs) in particular have always been considered a low-priority option for analgesia. The main scope of this article is to review the current literature on pain management specifically on the role of PNBs in trauma patients at risk of ACS in the ED and highlight guidelines and recommendations for safe clinical practice.

## 2. Trauma Patients in the ED

Trauma is a Greek word “τραύμα” that means wound. It should be pointed out that trauma patients in the ED form one of the most heterogeneous groups of patients. They cannot be strictly categorized by gender, age, human race, preexisting pathology, socioeconomic status, occupational diseases, geographic regions and type of injury. The reason for this heterogeneity is that trauma could affect everyone at any time, but potentially in a different way. Trauma involves a broad spectrum of incidents: wounded soldiers during military operations, motor vehicle accidents, bicycle strikes or collisions, diving accidents, falls from height and domestic violence or abuse incidents; moreover, it affects all age groups. Orthopedic injuries are mostly involved in trauma patients. From the beginning of the combat operations of the United States Army in Iraq, the military service members who were wounded suffered not only from injuries at the extremities but even from amputations. Among United States soldiers wounded in Iraq, during an 8-month period in 2003, 42% had suffered orthopedic injuries [1].

The whole medical approach should always include adequate pain management. In the context of the EDs, not only at central teaching hospitals but at the peripheral and rural ones as well, trauma patients can receive advanced analgesia care in well-organized settings that are adequately equipped. The practice of regional anesthesia as part of multimodal analgesia should be performed in a constantly increasing number of EDs [2].

Despite specific characteristics and contrasts, one common element that all trauma patients share is pain. It is important to emphasize that pain is the main reason that leads patients to ask for medical help. It is estimated that 70% of ED patients are undertreated for pain or not treated at all, and 60% of ED patients experience pain that is more intense at discharge than at admission [2]. Additionally, the number of patients who actually receive pain management while admitted to the ED is low, as less than 50% of them will be treated for pain [2]. In this particular group of patients, pain management is of crucial importance. Pain was introduced as the fifth vital sign (P5SV) in 1995, by James Campbell [3]. Pain that is not properly treated has a negative impact not only on patients but on the entire health system as well. Some of the implications of under- or mistreated pain are reduced physical activity and poor quality of life, an inability to rest and sleep properly, increased morbidity, the risk of chronic pain and increased economic costs due to a prolonged length of stay and readmission [4]. The negative aspects of inadequate pain management have a direct and detrimental impact on the overall patients’ conditions of life. The practice of the ATLS (Advanced Trauma Life Support) algorithm is an extremely important help that has significantly contributed to the resuscitation and management of trauma patients. The importance of pain management in trauma is emphasized by the suggestion to include the first letter of the word pain “P” in the ATLS algorithm [5]. Along these lines and after the initial resuscitation of the patient, a secondary evaluation will include pain management under specific guidelines so that the analgesic process will be guaranteed, though without impeding the resuscitation process [5].

## 3. History of Acute Compartment Syndrome, Pathophysiology, Diagnosis and Management

Acute compartment syndrome (ACS) is an emergency situation that requires immediate surgical intervention. It is impressive and educative to track down the pathway of ACS diagnosis and management over the years. Ancient Greeks had already recognized this entity and an early mention was found in the writings of Hippocrates, the Greek doctor of the 5th century BC known as the father of medicine [6,7]. ACS was initially described as an imbalance of the humors of the human body (black and yellow bile, phlegm and blood). It is a pathological condition that causes pain, pressure in the affected area, fever, empyema, orthopnea, cough and contraction of the corresponding veins. The treatment consisted of warm compresses, herbal medicines, suction cups, phlebotomy and skin incisions. The recommended surgical management was described to be a single and fast incision with minimal pain for the patient. All of these data suggest that ACS has been an issue of concern among physicians since the early days of medicine.

Medicine is constantly evolving, and new concepts are constantly introduced. In the 19th century, ACS was first described in 1881 by Volkmann [8]. Richard von Volkmann was a legendary surgeon with military experience and was considered a leader in orthopedic surgery [9]. He described that a hand deformity in the pediatric population could be attributed to ischemic muscle contraction and myonecrosis and launched the 5 ‘P’s’ of compartment syndrome (pain, pallor, paresthesia, paralysis and pulselessness) [8]. Volkmann also presented the antiseptic technique in his country, Germany [9]. Volkmann sent his assistant to Lister’s clinic, as Lister developed, in 1867, prophylactic measures for the prevention of wound infections. After his return, the first trials were successful in healing without pus or fever. Later, in 1874, Volkmann delivered a lecture about the antiseptic occlusive bandage, and this led to his notoriety [9]. In the next century, in 1949, Carter gave a clear and detailed definition of the syndrome [10,11].

**Figure 1:**
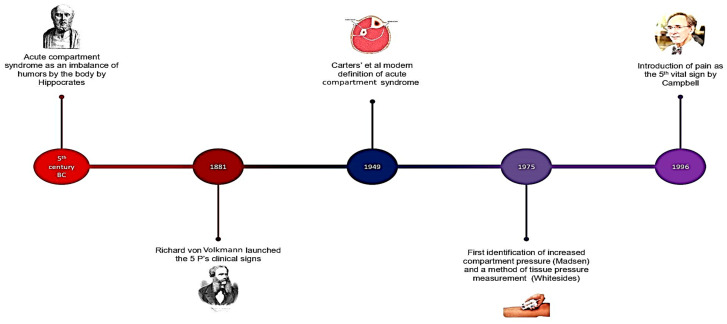
History of acute compartment syndrome [11].

Subsequently, in 1975, Matsen was the first to talk about increased compartment pressure and its correlation with the underlying mechanism of acute compartment syndrome [12,13]. Whitesides was the first to talk about a method of actually measuring that pressure (Figure 1) [12,14].

ACS is characterized by increased pressure in a closed anatomical compartment that leads to impaired vascularization and consequently to irreversible damage of the tissues. Data collected from the Royal Infirmary of Edinburgh of all patients visiting the orthopedic trauma unit in the time period of 1988–1995 with a diagnosis of ACS showed that 164 patients with a mean age of 32 years were recorded, and the annual incidence was 0.7–7.3/100,000 [15]. Young patients with an increased muscle mass are more likely to suffer from this syndrome in comparison to older ones with muscle atrophy. It is important to mention that it may take days for the syndrome to occur, and it is not always present within the first 48 h after injury [16]. The pathophysiology is rather complex. Increased pressure in a restricted compartment due to increased volume or decreased dimensions of the compartment leads to compromised vascularization that can induce ischemia and necrosis [17]. It is interesting and useful to know that ACS in a military environment occurs not only after trauma but even after crystalloid overloading during resuscitation.

An early and correct diagnosis is of major importance for the salvage of the affected area. There is also one more issue that is vitally important and that is the medico-legal risk. It is shown that incorrect or delayed diagnosis was responsible for 23% and 32% of legal claims, respectively, and compensation was USD 574,680 per cause [17]. The cornerstone of a correct and early diagnosis is based on constant vigilance. An evaluation of the signs and symptoms, clinical examinations and the use of diagnostic tools and/or devices should be used in order to form a diagnosis.

Pain has been believed to be one of the most important symptoms for the diagnosis of acute ACS. However, this belief is rather controversial, as the sensation of pain is subjective and varies among individuals. There are also cases when patients may be unable to communicate due to language barriers, but nowadays, fortunately, health care environments have useful available tools to overcome them, such as online translation sites. Another reason of major importance that patients cannot communicate their pain is their clinical status at the time of their admission to the hospital, and as a consequence, ethical and legal issues may arise in the emergency setting. Informed consent by patients is not always possible due to various conditions. These conditions include emotional tension, agitation, the consumption of substances, extreme ages, an altered level of consciousness, a lack of any authorized consent and information on allergies and preexisting nerve damage.

Additionally, pain may not be present due to progressed ischemic injury [18]. Traditionally, six signs and symptoms have a high suspicion index for ACS, with cold being the sixth element added to Volkmann’s five ‘Ps’ [19]. These symptoms and signs consist of pain, pallor, paresthesia, pulselessness, paralysis and of course cold. It seems though that the absence of signs and symptoms is more accurate to exclude a diagnosis of ACS rather than their presence confirming a diagnosis because of low sensitivity (13–19%) but high specificity (97–98%) [20,21].

A clinical examination is of major importance, and it should be focused on recognizing the wood-like feeling, which is indicative of ACS, when comparing the symptoms to the non-affected corresponding area and when the measurement of pressure in the compartment is of concern. Conditions that predispose patients to ACS include high-energy traumatic injuries accompanied by long bone fractures, the prolonged use of high-pressure tourniquets and patient malpositioning during surgery [18]. Measurement of the pressure in the affected compartment is not typically used although it is considered the gold standard method for the diagnosis of the syndrome [12] Normal intra-compartmental pressure (ICP) can be measured with various methods and devices, and it is important to underline that a single measurement within normal ICP limits (0–8 mmHg) cannot guarantee the absence of the syndrome [18].

Once the syndrome is diagnosed, the treatment of choice is surgical decompression and early fasciotomy with a constant evaluation of the patient’s clinical condition, monitoring, fluid management and hemodynamic stabilization [18].

## 4. Compartments of Interest at the Extremities

**Figure 2:**
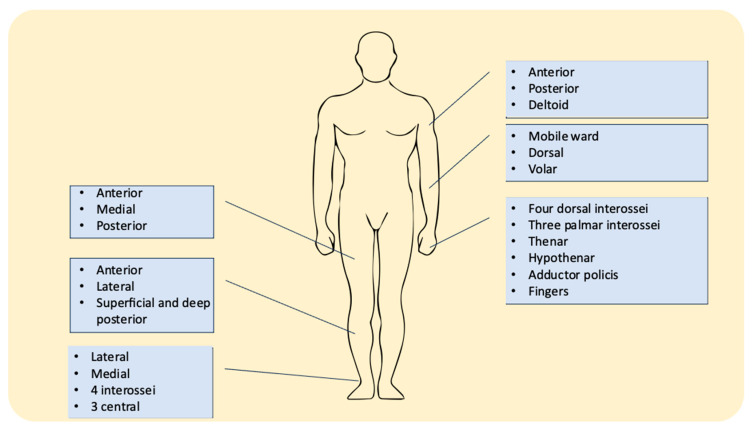
Compartments of interest at the extremities.

The upper and lower extremities are a major concern of orthopedic trauma patients with the tibia highly involved at a percentage of 36% of all cases in a large trauma center [15,22]. Understanding the anatomy of the involved areas is fundamental for the physician in order to plan the ideal pain management protocol and select the most appropriate PNB. The compartments involved are described in the literature, though with slight differences (Figure 2) [22,23,24].

Upper extremities

Upper arm: anterior, posterior, deltoid (3 compartments)

Forearm: mobile ward, dorsal, volar (3 compartments)

Hand: four dorsal interossei, three palmar interossei, thenar, hypothenar, adductor pollicis (10 compartments)

Fingers

Lower extremities

Thigh: anterior, medial, posterior (3 compartments)

Calf: anterior, lateral, superficial and deep posterior (4 compartments)

Foot: medial, lateral, four interossei, three central (9 compartments).

## 5. Guidelines and Recommendations

The practice of ultrasound-guided RA is of major importance in Emergency Medicine (EM). Trauma patients suffering from acute pain are often in critical condition and hemodynamically unstable. The benefits of RA and PNBs, in particular, are numerous as they offer a satisfying analgesic result, have minimum impact on a patient’s hemodynamic profile and help avoid the side effects of systemic opioid analgesics. Their role in combating the widespread epidemic opioid crisis is vital.

According to the relevant literature, it has been suggested that specific LA characteristics against edema and inflammation might be useful in avoiding ACS [18,25,26]. Due to their anti-inflammatory properties, they have effects on the pathway of the inflammatory cascade and on the synthesis and release of the mediators involved in the inflammatory process [25]. Their effects on capillary hyperpermeability and the formation of edemas induced by inflammation are based on properties like the inhibition of histamine release [25,27]. Four local anesthetics amides and esters, lidocaine, bupivacaine, mepivacaine and procaine, down-regulate the tumor necrosis factor-alpha (TNF-a) secreted by the lipopolysaccharide-induced response of leucocytes in human blood samples [26].

PNBs within the context of multimodal analgesia are of vital importance for the resuscitation of patients. Therefore, all EDs should have protocols and adequately trained staff to treat pain immediately and efficiently in order to provide the most optimum conditions for good outcomes [2]. The absence of standard protocols for training and education has led to great variability in ultrasound-guided PNB utilization in an ED setting. Since different EM educational programs are applied in different centers, the medical background of EM physicians is heterogeneous. There is always the risk of undertreatment and the onset of complications, especially when there are no standard protocols.

Hence, a curriculum for ultrasound-guided regional anesthesia has been created specifically for EM physicians (Table 1) [28]. A total of 13 experts in the field, from different countries and different centers, selected and suggested 178 elements which included 68 techniques and 110 background elements. The techniques were categorized by anatomic areas. Through the Delphi method, consensus was reached on 65 elements and 10 techniques. Departments are therefore invited to organize their practice and education based on this curriculum.

The techniques include the following:

Interscalene brachial plexus block, supraclavicular brachial plexus block, radial nerve block at the level of the forearm, median nerve block at the level of the forearm, ulnar nerve block at the level of the forearm, serratus anterior plane block, fascia iliaca plane block, femoral nerve block, popliteal sciatic nerve block and posterior tibial nerve block. As far as the background knowledge elements are concerned, they include benefits for the patient and the provider, risks, indications, contraindications, local anesthetics, sterile techniques, ultrasound skills, procedural skills and educational resources.

While writing the present review and according to our knowledge, there was no published randomized trial to compare the effects of PNBs in patients at risk for ACS with studies performed with no PNBs at all.

Several anesthesia societies have proposed guidelines on the use of PNBs and ACS. The Association of Anesthetists of Great Britain and Ireland recommends against the usage of adjuncts, in favor of selecting single-shot techniques or continuous PNBs with low concentrations of local anesthetics (LAs) and the avoidance of high-density nerve blocks in cases of lower leg trauma (Table 2) [20]. They emphasize the importance of post-injury and postoperative surveillance. The European Society of Regional Anaesthesia and Pain Therapy (ESRA) and the American Society of Regional Anesthesia and Pain Medicine (ASRA) Joint Committee Practice Advisory came to a consensus but for the pediatric population only. They suggest low doses of LAs, a restriction of doses and volume in high-risk compartments and the use of adjuvants with caution. Their recommendations are based on the fact that there is no data in the literature demonstrating that PNBs increase the risk of ACS or delay the diagnosis in children (Table 2) [29]. Eighteen experts chosen by the French physical and rehabilitation medicine (SOFMER) and Anesthesia and Intensive care (SFAR) societies proposed guidelines for peripheral motor nerve blocks with respect to physical and rehabilitation medicine (Table 2) [30]. According to these, PNBs could be performed in patients under anticoagulant therapy if it would be too risky for them to discontinue it. However, they underlined the need for vigilance and careful clinical evaluation after deep or superficial PNBs due to the risk of hematoma and ACS. Therefore, deep blocks under anticoagulant therapy should be avoided. Ultrasound techniques and an evaluation of international normalized ratio (INR) values are strongly recommended.

Administering PNBs in trauma patients mostly involves the upper and lower extremities. Regarding the upper extremities, a single block can cause the blockade of the extremity, while more blocks are needed in order to anesthetize the lower limb [8]. In the case of a tibia plateau for example, a saphenous nerve block can be performed when, at the same time, the compartments below the knee that receive innervation from the sciatic nerve remain unaffected by the blockade [8]. Following guidelines and recommendations are of great value in the context of the safe practice of PNBs in the anatomic areas of concern.

## 6. Discussion

RA and the risk of ACS have always been a matter of scientific debate among researchers, as there is a part that encourages the practice of PNBs and a part that is not in favor. The specialty of anesthesiology evolved significantly after War World II because mass casualties stimulated battlefield anesthesia, and the training programs bloomed, creating a reciprocal ironic relationship between war and anesthesiology. On the one hand, war helped the evolution of anesthesiology, and on the other hand, anesthesiology helped the victims of war [31]. The Iraq and Afghanistan wars further contributed to anesthesiology’s evolution and particularly to the evolution of regional anesthesia, mostly due to the widespread use of continuous PNBs on the battlefield [32]. Although, in the literature, it is also suggested that PNBs should be avoided in patients at high risk for ACS because they could be responsible for a delayed diagnosis of ACS, the PNB distribution area seems to have no relation to the area of ACS [18,33]. In order not to obscure the diagnosis of ACS, it is recommended to use an analgesic and not anesthetic dosage of LAs for a safer practice of PNBs [34].

Pain is beyond any doubt the main reason for ED visits among conscious patients. Trained staff and appropriate equipment are necessary for safe and early pain management. Site-specific analgesia protocols are important not only for providing high-quality analgesia but also in order to reduce overall opioid consumption. Consequently, the short- and long-term negative effects of opioids are reduced in order to achieve a minimum burden on the respiratory and cardiovascular systems and improve morbidity and mortality [35]. There is a need for multidisciplinary collaboration in the EDs, as regional anesthesia techniques are nowadays performed not only by anesthesiologists but by emergency physicians as well. In current clinical practice, several teaching methods are being applied, and various techniques are being performed at different EDs. The most popular choice in EM in the field of regional anesthesia is administering single-shot PNBs. However, the use of continuous PNBs with catheter placements in the ED would be valuable as well [35]. The advantage of continuous analgesia using continuous PNBs with peripheral nerve catheters should be strongly considered.

Patient care should always be the first priority of all health workers, and physicians coming from different fields should definitely communicate and decide together on the patient’s optimal treatment. However, it should be recognized that anesthesiologists are the experts in the field of pain management, and therefore their opinion on the analgesic treatment should be highly appreciated [20]. After all, according to the Declaration of Montreal, analgesia is a fundamental human right [36]. According to IASP (International Association for the Study of Pain), inadequate pain management all over the world leads to suffering that could have been avoided, and so in 2010, the association IASP proceeded with the declaration in order to assure human rights.

The combination of two or more drugs with different mechanisms of action is definitely of great help to trauma patients. The negative effects are minimized and there is no need to increase the dose of a single drug, consequently increasing possible complications and adverse side effects. Regional anesthesia techniques, systemic analgesia and non-pharmacological treatments are all part of the context of multimodal analgesia. It is also very important to notice that this approach can be applied at different steps of the trauma patient pathway, from the time of arrival at the ED and during the whole length of stay, according to the severity of the pain [37]. In this very special group of patients, PNBs are recommended as an important and indispensable part of the multimodal analgesic approach. Ultrasound techniques have contributed significantly to site-specific analgesia, targeted to provide a nerve blockade of the area of interest and only sparing pharmacological burden and avoiding a more generalized block involving even territories distant from the area of concern. A very strong advantage of the ultrasound-guided PNB techniques performed in the EDs is that in many anatomic areas, the target can be visualized without the need to reposition the patient, an action that in trauma patients could be devastating [38]. It is vital for the patients to have all the pain management they need and distance themselves from the negative feelings of distress, as it is very possible that all the distress that the patient can experience can activate the vicious cycle of trauma–pain–stress–feedback [37].

Peripheral nerve blocks are not contraindicated for patients at risk for ACS, as long as there is surveillance, and analgesic and non-anesthetic doses of local anesthetics are used. Additionally, as the nerve blockade involves a very specific anatomic area, the diagnosis of ACS in other anatomic areas where peripheral nerves are not blocked might be easier to make [39]. An observation that is rather interesting and worth mentioning is that studies in favor of RA in patients at risk for ACS are most commonly published in anesthesiology journals, whereas studies against the practice of RA in this special group of patients are published in journals in the field of surgery [18,40,41]. It should also be mentioned that the literature is frugal in randomized controlled trials in the field of regional anesthesia and the imminent risk of acute compartment syndrome [42]. The rational use of local anesthetics in doses and concentrations that will result in short-lasting blocks that are not dense is recommended [43]. It is important to highlight that vigilance is of major importance for the diagnosis of ACS, despite the analgesic approach that is chosen [44].

## 7. Conclusions

Adequate and immediate relief from acute traumatic pain is of vital importance for patients’ resuscitation in the emergency setting. The safe practice of PNBs, single shot or continuous, with analgesic and non-anesthetic doses of LAs, is of major importance in the field of multimodal analgesia. The risk of ACS should always be taken under serious consideration, but without compromising optimal pain management.

## 8. Future Directions

Future directions should include training using ultrasound regional techniques at all EDs, protocols based on the specific needs of each setting and teamwork aiming for the best because pain is a human right and must be respected.

## Figures and Tables

**Table 1:** Consensus on techniques.

Consensus on 10 Ultrasound-Guided Regional Anesthesia (UGRA) Techniques		
1.	Upper Extremity	Interscalene brachial plexus block, supraclavicular brachial plexus block, radial nerve block at the level of the forearm, median nerve block at the level of the forearm, ulnar nerve block at the level of the forearm.
2.	Trunk	Serratus anterior plane block.
3.	Lower Extremity	Fascia iliaca plane block, femoral nerve block, popliteal sciatic nerve block, posterior tibial nerve block.

**Table 2:** Anesthesia societies’ guidelines.

Anesthesia Societies’ Guidelines on the Use of Peripheral Nerve Blocks (PNBs) and Acute Compartment Syndrome (ACS)	
Association of Anesthetists of Great Britain and Ireland	The available literature suggests that the use of neuraxial or peripheral regional techniques resulting in dense blocks of long duration, significantly exceeding the duration of surgery, should be avoided. The available literature suggests that single-shot or continuous peripheral nerve blocks using lower concentrations of local anesthetic drugs without adjuncts are not associated with delays in diagnoses provided post-injury. Postoperative surveillance is appropriate and effective.
European Society of Regional Anaesthesia and Pain Therapy (ESRA) and the American Society of Regional Anesthesia and Pain Medicine (ASRA) Joint Committee Practice Advisory Consensus (for pediatric population only).	There is no current evidence that the use of regional anesthetics increases the risk for ACS or delays its diagnosis in children.
French physical and rehabilitation medicine (SOFMER) and Anesthesia and Intensive care (SFAR) societies [3].	In physical and rehabilitation medicine (PRM), in the practice of motor blocks, often distal and involving deep nerve trunks, the group of experts proposes to consider the risk of the consequences of the occurrence of a hematoma: minimal in the case of a superficial hematoma, and more significant in the case of a deep hematoma threatening a compartment syndrome. This risk must be taken into account in the benefit–risk balance.
